# Corrosion behavior of polar marine steel FH40 in simulated marine environments

**DOI:** 10.1039/d6ra08961e

**Published:** 2026-11-12

**Authors:** Liangliang Hu, Dongsheng Wang, Juan Zhao, Yitao Wang, Xuhui Gong, Penglong Ding, Xueting Chang

**Affiliations:** a College of Ocean Science and Engineering, Shanghai Maritime University Shanghai 201306 China xtchang@shmtu.edu.cn wangds@shmtu.edu.cn; b National Key Laboratory of Ship Structure Safety Wuxi 214082 China; c Luoyang Ship Material Research Institute Luoyang 471023 China

## Abstract

With the increasing strategic value of the Arctic Passage, corrosion behavior of polar marine steel in harsh environments has become a key issue for ship life-cycle reliability. This work systematically compares the corrosion behavior of FH40 polar marine low-temperature steel under three conditions: 20 °C immersion, 20 °C salt spray, and −60 °C immersion. Combined with EBSD characterization, the effects of environmental factors on the corrosion mechanism, product evolution, grain boundary characteristics, and dislocation density are elucidated. Results show that the corrosion mechanism is synergistically controlled by environment and temperature. Immersion at 20 °C is dominated by uniform dissolution with local pitting, while −60 °C immersion presents typical local pitting. In contrast, 20 °C salt spray exhibits crater-like morphology with internal accumulation and deep edge erosion, resulting from dry–wet cycling and Cl^−^ enrichment. EBSD analysis indicates that all samples are dominated by high-angle grain boundaries (HAGB). The salt spray sample shows the highest HAGB fraction (69.4%) and the highest grain-boundary density (0.42 µm^−1^), together with the smallest average grain size (7.11 µm), consistent with its highest corrosion rate. Electrochemical tests confirm that corrosion products provide no protective effect; instead, they accelerate substrate corrosion by forming oxygen concentration cells. Although the rust layer of the salt spray sample is approximately 3–4 times thicker than that of the immersion sample, its weight loss and corrosion rates are correspondingly 3–4 times higher, indicating no positive correlation between rust layer thickness and protective performance. Low temperature significantly inhibits corrosion kinetics. Compared with 20 °C, the corrosion rate and rust layer thickness at −60 °C decrease by approximately one order of magnitude, and grain boundary structure and dislocation density remain at low levels. This work addresses a critical knowledge gap in comparative study of immersion and salt spray corrosion of low-temperature marine steel, providing a basis for service life prediction and protective design of polar marine vessels.

## Abstract 2

Corrosion of polar steel FH40 is compared under 20 °C immersion, 20 °C salt spray and −60 °C immersion. Salt spray gives a thicker yet non-protective rust layer; −60 °C suppresses corrosion by an order of magnitude.

## Graphical abstract

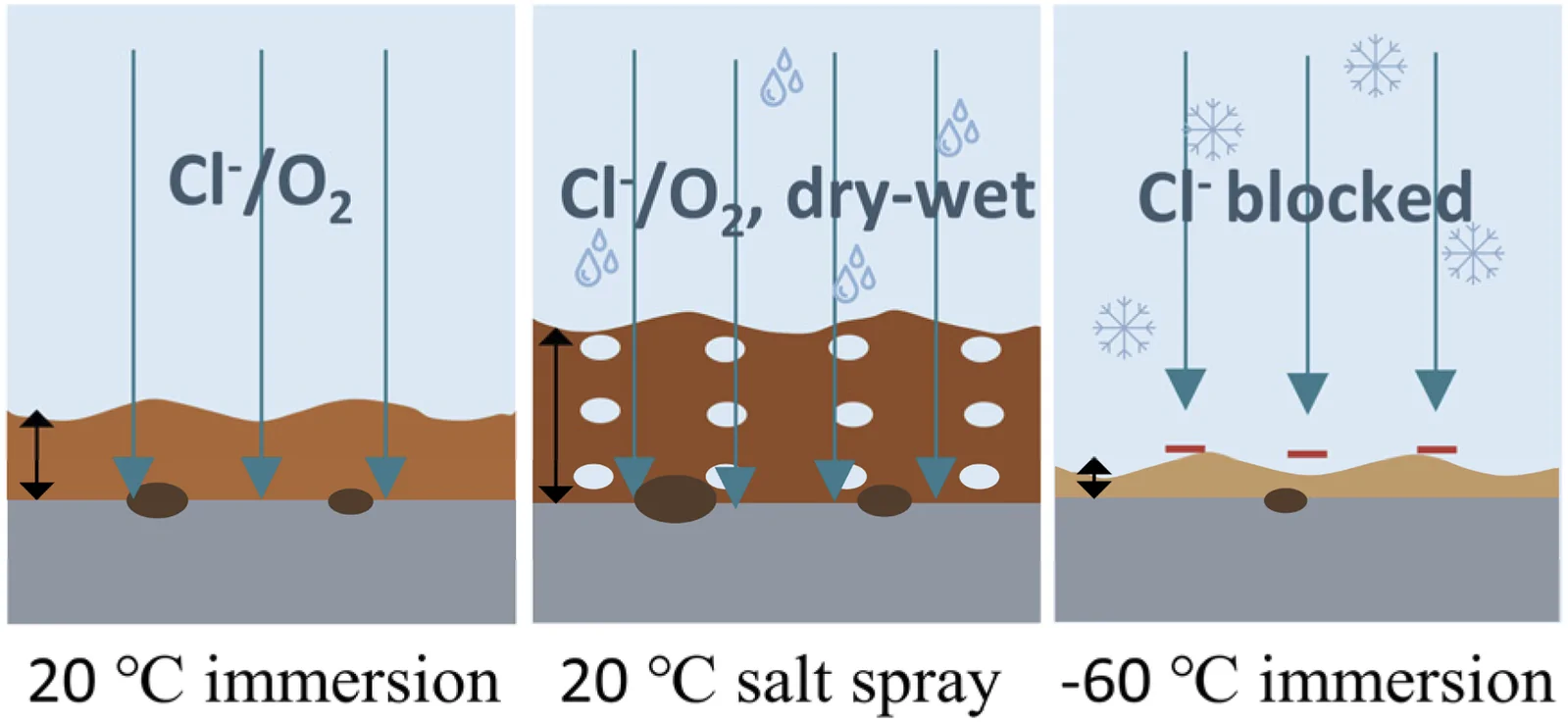

## 1 Introduction

With the accelerated global climate change, the area of the Arctic ice sheets has been continuously shrinking, and the strategic value and economic potential of the Arctic shipping route have become increasingly prominent. The undiscovered oil and natural gas reserves in the Arctic account for 13% and 30% of the global total, respectively,^1^ along with abundant reserves of strategic resources including rare earths and rare metals.^2–4^ Meanwhile, the regular navigation of the Arctic shipping route can greatly shorten the distance of the Asia-Europe shipping route, and significantly reduce shipping costs and carbon emissions, showing enormous shipping potential. Compared with traditional ships, polar ships operate continuously in harsh environments characterized by high humidity and salt spray concentrations. Regardless of whether the hull is immersed in seawater or exposed to the atmosphere, corrosion readily occurs, compromising structural integrity and posing significant risks to vessel safety and functionality. Therefore, the systematic study of the corrosion behavior of polar marine steel in marine environments has become a key scientific issue to ensure the reliability of polar ships throughout their service life.

Immersion and salt spray corrosion are two important accelerated corrosion test methods. The former simulates the continuous electrochemical erosion of the hull immersed in seawater for a long time, and the latter reproduces the intermittent salt deposition and dry–wet cycling in the high-humidity and high-salt spray atmosphere of the Arctic. There are essential differences between the two in terms of corrosion medium, oxygen supply mode and wetting mode. Numerous researchers have systematically compared the similarities and differences in corrosion behavior between immersion and salt spray conditions. Krawiec *et al.*^5^ conducted a comparative study on salt spray and immersion corrosion of Mg–Zn alloy and found that the corrosion products under salt spray were denser and more protective, while more CaCO_3_ was formed under immersion with more severe corrosion. For the study on the protective effect of corrosion products on the substrate, Zhang *et al.*^6^ studied the initial corrosion behavior of 7B04 aluminum alloy under acidic salt spray and acid immersion environments and found that corrosion products had an inhibitory effect on corrosion, and the corrosion product layer under salt spray environment was thicker with stronger corrosion inhibition ability. Song *et al.*^7^ conducted a comparative study on AM30 magnesium alloy and found that the corrosion products in the immersion environment had higher solubility and poorer protective performance, while the corrosion products in the salt spray environment were more stable with slower pitting development. Martin *et al.*^8^ believed that the accumulation of corrosion products would promote continuous corrosion. In terms of corrosion mechanism, Martin *et al.*^8,9^ studied magnesium alloys and found that under immersion conditions, pitting was extensive in both number and size, although the pit depth decreased with time, dominated by combined general and pitting corrosion; under salt spray conditions, pitting corrosion was more readily induced, producing fewer but deeper pits with larger area and volume, dominated by combined pitting and intergranular corrosion. Walton *et al.*^10^ also studied magnesium alloy and held that immersion was dominated by general corrosion and intergranular corrosion, while salt spray was more conducive to local deep corrosion. Controversy persists regarding the relative corrosion rates under the two conditions. Most studies report higher rates under salt spray than under immersion,^11–16^ with a maximum difference of 9.5 times;^17^ however, several studies have found immersion to be more aggressive.^5,7–9^ In terms of low-temperature immersion experiments, Wen *et al.*^18^ systematically studied the immersion corrosion behavior of Q420 low-alloy steel under four conditions: constant temperature of −30 °C, 0 °C, 25 °C and freeze–thaw cycling of 25/−30 °C, and found that there was an obvious electrochemical corrosion process under low-temperature freezing conditions. Fu *et al.*^19^ confirmed that completely frozen NaCl solution still retained conductivity and corrosivity.

The comparative studies reviewed above have focused mainly on aluminium alloys, magnesium alloys and related light-metal systems. For structural steels, and in particular for polar marine low-temperature steels, a systematic comparison of immersion *versus* salt-spray corrosion—especially extending to cryogenic temperatures relevant to Arctic service—remains largely unexplored. Accordingly, the present work takes the polar marine low-temperature steel FH40 as the research object and systematically compares the corrosion products, corrosion rates, corrosion mechanisms and product protectiveness under immersion and salt-spray environments at 20 °C and −60 °C. This study addresses a critical knowledge gap in the corrosion behaviour of low-temperature steels and provides a key basis for the service-life prediction and green protective design of polar ships.

## 2 Experimental methods

The material used in this work was FH40 marine low-temperature steel. The steel was sectioned by wire electrical discharge machining (WEDM) into samples of 10 mm × 10 mm × 3 mm for microstructure observation, characterisation and electrochemical tests, and samples of 30 mm × 50 mm × 3 mm for mass-loss tests. Both sizes of samples were treated with the same grinding sequence and drying and cleaning method: ground sequentially with 180, 400, 800, 1200, 1500, and 2000 grit silicon carbide sandpapers, followed by polishing to a mirror finish using a 2.5 µm diamond suspension. The samples were then ultrasonically cleaned in ethanol, dried in an oven, and stored in a desiccator prior to use. Fig. 1 shows the metallographic structure after etching in 4% volume fraction nitric acid alcohol solution for 10 s. It can be seen from the figure that FH40 steel is composed of polygonal ferrite (F) and pearlite (P), the ferrite is in an irregular polygonal shape, and the pearlite is distributed between ferrite in the form of black fine needles.

**Fig. 1:**
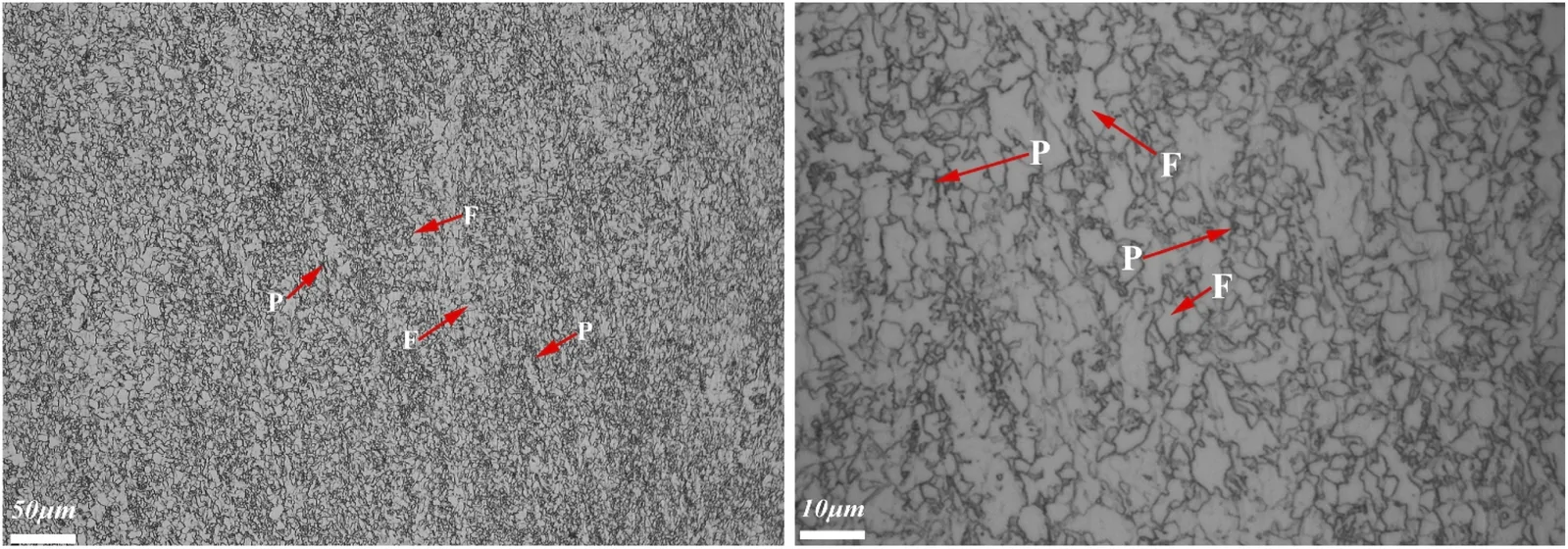
Metallographic structure of marine low-temperature steel FH40.

Due to the essential differences between salt spray test and full immersion corrosion test in corrosion mechanism, medium renewal frequency, oxygen diffusion rate and other aspects, different standard cycles are usually adopted for the two in engineering practice. In this study, the standard cycles of each method were followed respectively to better simulate the typical assessment methods of the two corrosion environments in actual engineering applications. The neutral salt spray corrosion test of simulated seawater at 20 °C was carried out according to ASTM G85 (ref. 20) for 1000 h, and the immersion corrosion test of simulated seawater at 20 °C and −60 °C was carried out according to ASTM G31 (ref. 21) and GB 10124-88 (ref. 22) for 1200 h. This duration was chosen based on standard practices at multiple corrosion testing facilities and provides adequate representativeness for simulating medium-term corrosion behavior.^23–25^ Under the 20 °C test condition, the simulated seawater was renewed every 7 days, and the simulated seawater used in both the salt-spray and immersion tests was a 3.5 wt% NaCl solution. Since the simulated seawater was completely frozen at −60 °C, the samples at this temperature were maintained continuously at a constant −60 °C throughout the entire 1200 h exposure period, and no freeze–thaw cycling occurred. Corrosion products were removed following GB/T 16545,^26^ and the resulting mass loss was used to evaluate the corrosion resistance of the steel plate. The corrosion rate C (mm a^−1^) was calculated according to eqn (1):1
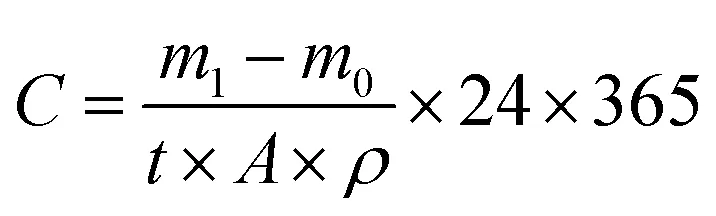
where: *C* is the corrosion rate (mm a^−1^), *m*_1_ and *m*_0_ are the weight of the sample after and before the test (g), respectively, *ρ* is the density of the sample (g mm^−3^), 0.00788 g mm^−3^ for FH40, *A* is the exposed area of the sample (1500 mm^2^ in this work), and *t* is the exposure time (h).

The electrochemical samples were welded to copper wires on their back faces and encapsulated in epoxy resin, leaving an exposed working area of 1 cm^2^. After receiving the same surface pretreatment as the mass-loss coupons, the electrochemical samples were exposed in parallel with the mass-loss coupons in the same chamber under identical conditions. Upon completion of the predetermined exposure period, each sample was directly transferred to a three-electrode cell containing the simulated seawater. Prior to each measurement, the open circuit potential (OCP) was allowed to stabilize for 30 min; electrochemical impedance spectroscopy (EIS) and the polarization curve were then measured in sequence with the corrosion products retained on the sample surface. Subsequently, the surface corrosion products were removed in accordance with GB/T 16545,^26^ and EIS and polarization measurements were repeated following the same procedure in the cleaned state. The simulated seawater was renewed between consecutive measurements to eliminate interference from the preceding test. In addition, an unexposed baseline sample (exposure time = 0 h), designated as the “initial” sample, was prepared with the identical surface pretreatment and tested directly to characterize the as-prepared surface state as a reference for the exposed samples.

All electrochemical measurements were performed in simulated seawater using a CHI660e electrochemical workstation in a three-electrode configuration, in which the sample served as the working electrode, a platinum sheet as the counter electrode, and a saturated calomel electrode (SCE) as the reference electrode. EIS spectra were collected over a frequency range of 10^5^ to 10^−2^ Hz, and the polarization curves were scanned from −1 V to +0.5 V with respect to the OCP, *i.e.* [OCP − 1 V, OCP + 0.5 V], at a scan rate of 1 mV s^−1^. Since the polarization scan irreversibly alters the sample surface and therefore cannot be repeated on the same sample, each electrochemical measurement under a given condition was performed once on an individual sample (*n* = 1); the corresponding results should be regarded as representative/indicative data.

A ZEISS Sigma 300 scanning electron microscope (SEM) equipped with an energy dispersive spectrometer (EDS) was used to characterize the corrosion morphology before and after removing the corrosion products. A Bruker D8 Advance X-ray diffractometer (XRD) was used to analyze the composition of corrosion products with a copper target, a scanning range of 5–90° and a scanning speed of 2° min^−1^. After removing corrosion products, electron backscatter diffraction (EBSD, EDAX Hikari Plus) was used to characterize the microstructures to obtain the crystallographic information of the steel matrix after corrosion. Data processing was performed using AZtecCrystal software. A Bruker Contour GT-1 white light interferometer was used to observe the three-dimensional morphology of the sample surface after removing the corrosion products, and extract the corrosion pit depth distribution and surface roughness information.

## 3 Results and discussion

### 3.1 Macroscopic morphology analysis

Fig. 2 shows the macroscopic morphology of marine low-temperature steel FH40 after corrosion in artificial seawater immersion and neutral salt spray environments. The macroscopic morphology of the corrosion products under the two environments exhibited distinctly different physical characteristics. It can be seen from Fig. 2a and c that the corrosion products under 20 °C immersion environment are reddish brown with a delicate and dense surface, while under salt spray conditions, the rust layer appeared as a mixture of black-brown and reddish-brown phases, and the surface of corrosion products is relatively rough. As shown in Fig. 2b and d, following corrosion product removal, the substrate surface under immersion conditions exhibited dense pitting and was notably smoother, whereas that under salt spray conditions displayed a more pronounced gully-like morphology and appeared considerably rougher. It can be seen from Fig. 2e that the corrosion products under −60 °C immersion environment are black brown, and the sample surface shows local corrosion characteristics.

**Fig. 2:**
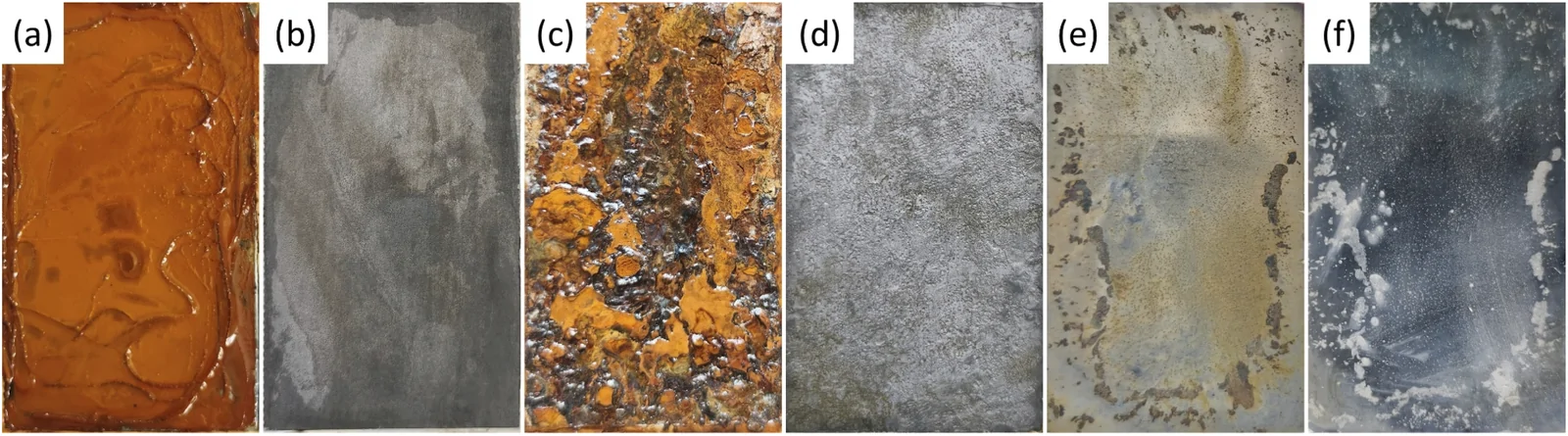
Macroscopic morphology of FH40 after immersion and salt spray corrosion: (a, c and e) with corrosion products retained; (b, d and f) after corrosion product removal; (a and b) 20 °C immersion, (c and d) 20 °C salt spray, (e and f) −60 °C immersion.

Table 1 shows the mass loss and corrosion rate of FH40 after immersion and salt spray corrosion. The average corrosion rate under 20 °C immersion environment calculated by eqn (1) is 0.1187 mm a^−1^, and that under 20 °C salt spray environment is 0.4340 mm a^−1^, about 4 times the former, indicating that active dissolution of the steel was more pronounced under salt spray conditions. The average corrosion rate under −60 °C immersion environment is 0.0126 mm a^−1^, which is an order of magnitude different from that under 20 °C immersion environment. Although the frozen NaCl solution retained conductivity and corrosivity at low temperatures, the corrosion rate was significantly reduced compared with that at room temperature.

**Table 1:** Mass loss and corrosion rate of FH40 after immersion and salt spray corrosion

Corrosion type	Sample no.	Initial weight (g)	Weight with corrosion products (g)	Weight after corrosion product removal (g)	Corrosion rate (mm a^−1^)
20 °C immersion corrosion	1	33.5615	33.4942	33.3744	0.1155
	2	32.6097	32.5380	32.4155	0.1199
	3	32.1283	32.0676	31.9323	0.1208
Average		32.7663	32.6999	32.5741	0.1187
20 °C salt spray corrosion	1	31.0287	31.3514	30.4479	0.4304
	2	32.3771	32.5925	31.7996	0.4280
	3	32.4096	32.6663	31.8108	0.4437
Average		31.9384	32.2034	31.3528	0.4340
−60 °C immersion corrosion	1	34.9479	34.9400	34.9317	0.0100
	2	33.4955	33.4867	33.4776	0.0111
	3	35.7088	35.6956	35.6818	0.0167
Average		34.7174	34.7074	34.6970	0.0126

### 3.2 Microscopic morphology analysis

Fig. 3 shows the microscopic morphology of FH40 after immersion and salt spray corrosion. The corrosion products formed under 20 °C immersion (Fig. 3a and b) exhibited a cluster-like cauliflower-like particulate morphology with a small amount of flaky protrusions locally on the surface. The microscopic morphology of corrosion products generated by 20 °C salt spray corrosion (Fig. 3d–f) shows flaky, rod-like and granular shapes simultaneously, which may be due to the differences in local microenvironments during the corrosion process. The corrosion products generated under −60 °C immersion (Fig. 3j and k) exhibited acicular and flaky morphologies. It can be seen from Fig. 3l that the −60 °C immersion sample presents a typical localized pitting morphology. Compared with the samples under 20 °C immersion and salt spray conditions (Fig. 3c and g), the former has fewer and smaller corrosion pits with significantly lighter corrosion degree.

**Fig. 3:**
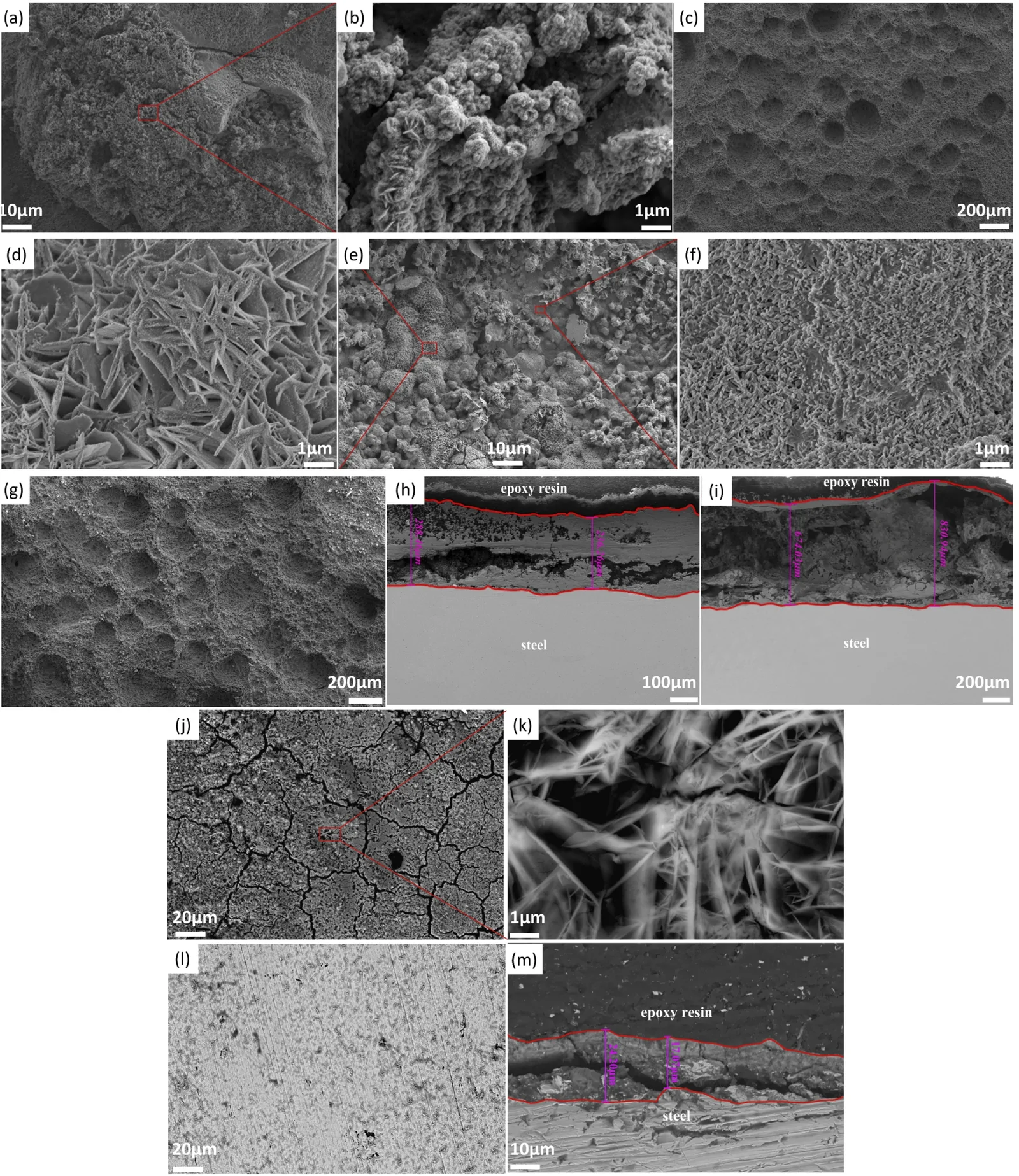
Microscopic morphology of FH40 after immersion and salt spray corrosion: (a and b) 20 °C immersion, (c) 20 °C immersion after corrosion product removal, (d–f) 20 °C salt spray, (g) 20 °C salt spray after corrosion product removal, (h) 20 °C immersion cross-section, (i) 20 °C salt spray cross-section, (j and k) −60 °C immersion, (l) −60 °C immersion after corrosion product removal, (m) −60 °C immersion cross-section.

The thickness of corrosion products of the sample under 20 °C immersion environment is about 236.26–288.46 µm (Fig. 3h), and that under 20 °C salt spray environment is higher, about 674.03–830.94 µm (Fig. 3i), while that under −60 °C immersion was only approximately 17.07–24.20 µm (Fig. 3m), which is an order of magnitude different from the value under 20 °C immersion environment. This result indicates that the extreme low-temperature environment can not only significantly reduce the corrosion rate of FH40 steel, but also significantly reduce the thickness of its corrosion product layer. As shown in Fig. 3i and h, the inner layer of the corrosion products on the salt spray sample was more porous and loose compared with that on the immersion sample. Although the thickness of corrosion products of the salt spray sample is 3–4 times that of the immersion sample, its mass loss and corrosion rate are 3–4 times higher than those of the immersion sample. These results demonstrate that the protective efficacy of corrosion products and the corrosion resistance of the steel cannot be inferred solely from their thickness.

### 3.3 Corrosion pit morphology analysis

Fig. 4 shows the corrosion pit morphology of FH40 after immersion and salt spray corrosion photographed by a white light interferometer.

**Fig. 4:**
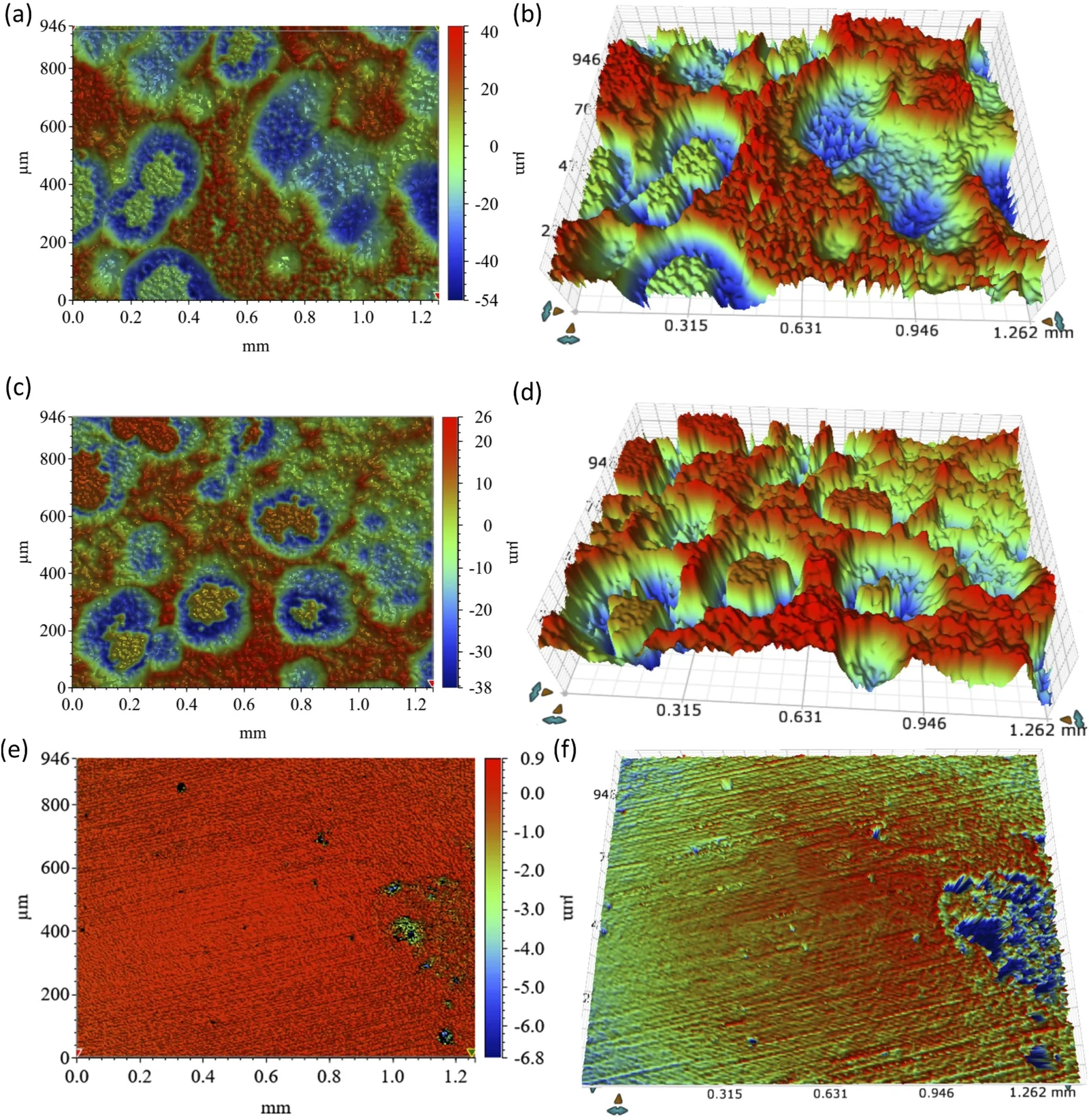
Corrosion pit morphology of FH40 captured by white-light interferometry: (a and b) 20 °C immersion, (c and d) 20 °C salt spray, (e and f) −60 °C immersion.

It can be seen from Fig. 4a–d that the corrosion pits of both 20 °C immersion sample and 20 °C salt spray sample have the morphological characteristics of high middle and low surrounding, which may be due to the redistribution and deposition of corrosion products or uneven local dissolution of the substrate. By comparing Fig. 4b and d, it is found that the “mouth-bottom” height difference of the salt spray sample is significant, while that of the immersion sample is almost gentle, indicating that immersion corrosion was dominated by uniform dissolution and localized pitting, whereas the salt spray sample exhibited a crater-like morphology with internal accumulation and deep edge etching. It can be seen from Fig. 4e and f that the immersion sample at −60 °C shows obvious localized pitting characteristics, and the depth and diameter of its pitting pits are smaller than those of the 20 °C immersion sample, with significantly weaker overall corrosion degree.

### 3.4 Corrosion electrochemical analysis

Fig. 5 shows the polarization curves of FH40 in immersion and salt spray corrosion environments. The corrosion potential (*E*_corr_), corrosion current density (*i*_corr_), anodic Tafel slope *β*_a_ and cathodic Tafel slope *β*_c_ fitted by the Tafel extrapolation method are shown in Table 2. In the Tafel region of the anodic polarization curves (Fig. 5), at the same potential the salt-spray and immersion samples with corrosion products retained exhibit higher current densities than the corresponding samples with corrosion products removed, indicating that the corrosion products formed under both salt-spray and immersion conditions provided no protection to the substrate but rather accelerated corrosion. It can be seen from Table 2 that removal of the corrosion products led to a significant decrease in corrosion current density for both immersion and salt spray samples, which further indicates that the corrosion products of FH40 in immersion and salt spray environments have no protective effect on the substrate. In addition, after removal of the corrosion products, the *i*_corr_ values of the 20 °C and −60 °C immersion samples were 11.210 and 7.578 µA cm^−2^, respectively, which are comparable to or slightly lower than that of the initial sample (12.070 µA cm^−2^); the 20 °C immersion sample is only about 7% lower than the initial sample. In contrast, the 20 °C salt-spray sample (16.530 µA cm^−2^) remained considerably higher than the initial sample. These results indicate that, after removal of the corrosion products, the corrosion current density of the immersion samples decreased to the initial level or even below, whereas that of the salt-spray sample remained significantly higher, suggesting that the corrosion damage on the surface of the salt-spray sample was not effectively eliminated even after product removal.

**Fig. 5:**
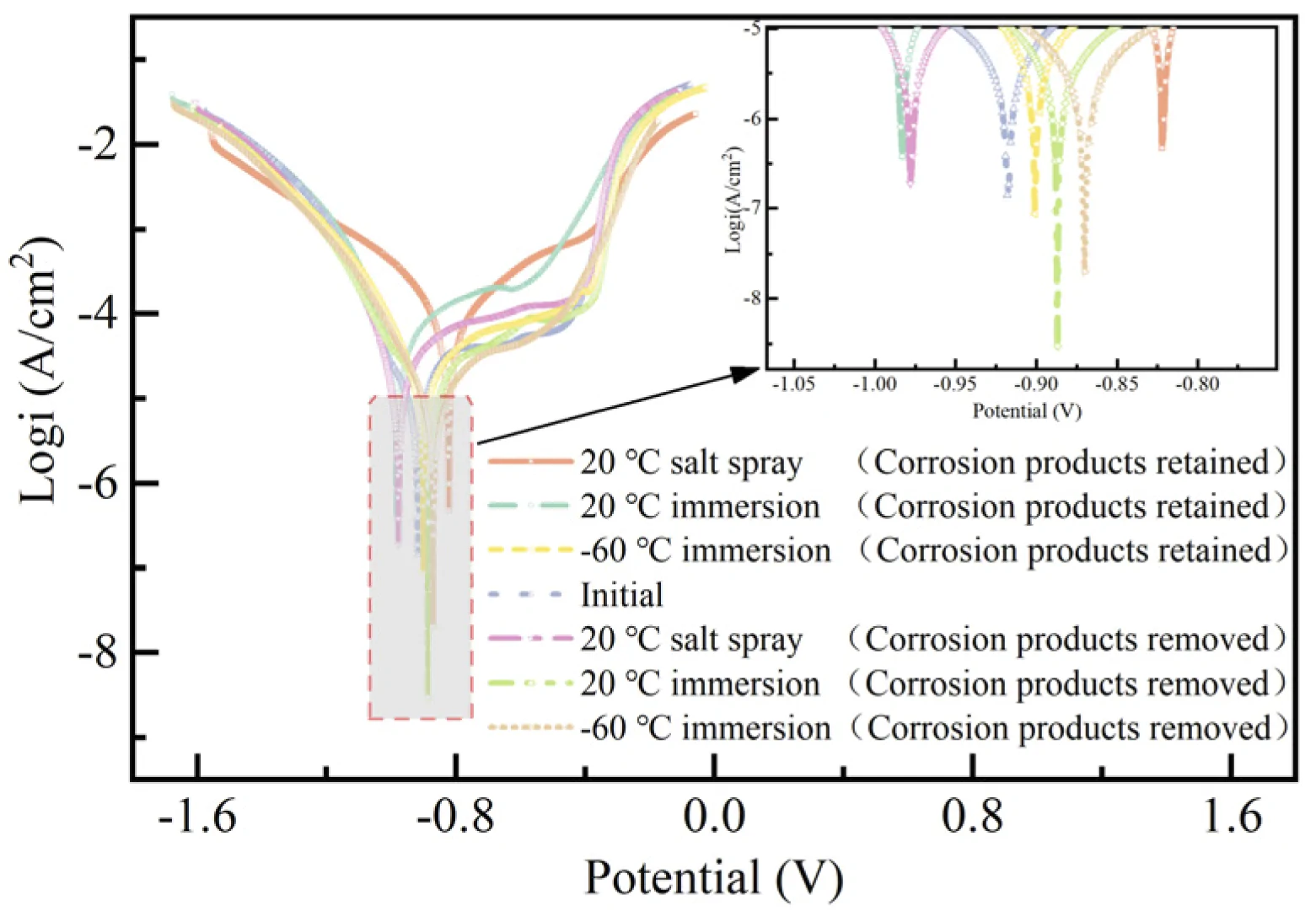
Polarization curves of FH40 after immersion and salt spray corrosion.

**Table 2:** Parameters fitted by Tafel extrapolation method

Sample type	*E*_corr_ (V)	*i*_corr_ (µA cm^−2^)	*β*_a_	*β*_c_	Note
Initial	−0.918	12.070	0.303	0.128	
20 °C immersion	−0.983	33.780	0.239	0.114	Corrosion products retained
	−0.887	11.210	0.240	0.208	Corrosion products removed
20 °C salt spray	−0.822	49.150	0.210	0.173	Corrosion products retained
	−0.978	16.530	0.235	0.111	Corrosion products removed
−60 °C immersion	−0.901	14.149	0.249	0.126	Corrosion products retained
	−0.870	7.578	0.225	0.138	Corrosion products removed

Fig. 6 shows the EIS results of FH40 after corrosion in immersion and salt spray corrosion environments, fitted with the equivalent circuit shown in Fig. 7, where *R*_s_ is the solution resistance, *R*_ct_ is the charge transfer resistance, CPE is the constant phase element (CPE_dl is the double layer capacitance, CPE_f is the film capacitance), and *W* is the Warburg impedance. The partial parameters of the fitted circuit are shown in Table 3, CPE_Y_0_ is the admittance constant, and CPE_n is the dispersion coefficient. It can be seen from Fig. 6a that the corrosion resistance of immersion and salt spray samples after removing the corrosion products is higher than that of the samples with corrosion products retained, and both are lower than that of the initial steel sample. The corrosion products formed under both salt spray and immersion conditions offered no protective benefit to the substrate.

**Fig. 6:**
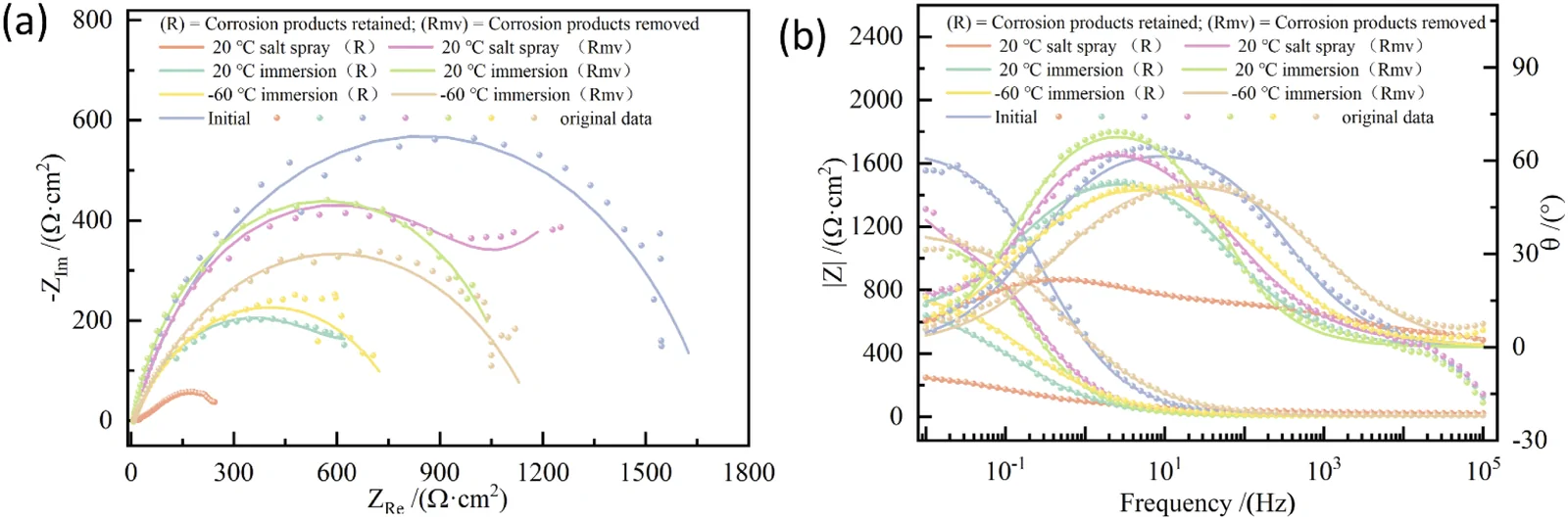
Electrochemical impedance spectra of FH40 after immersion and salt spray corrosion: (a) Nyquist plot, (b) Bode plot.

**Fig. 7:**
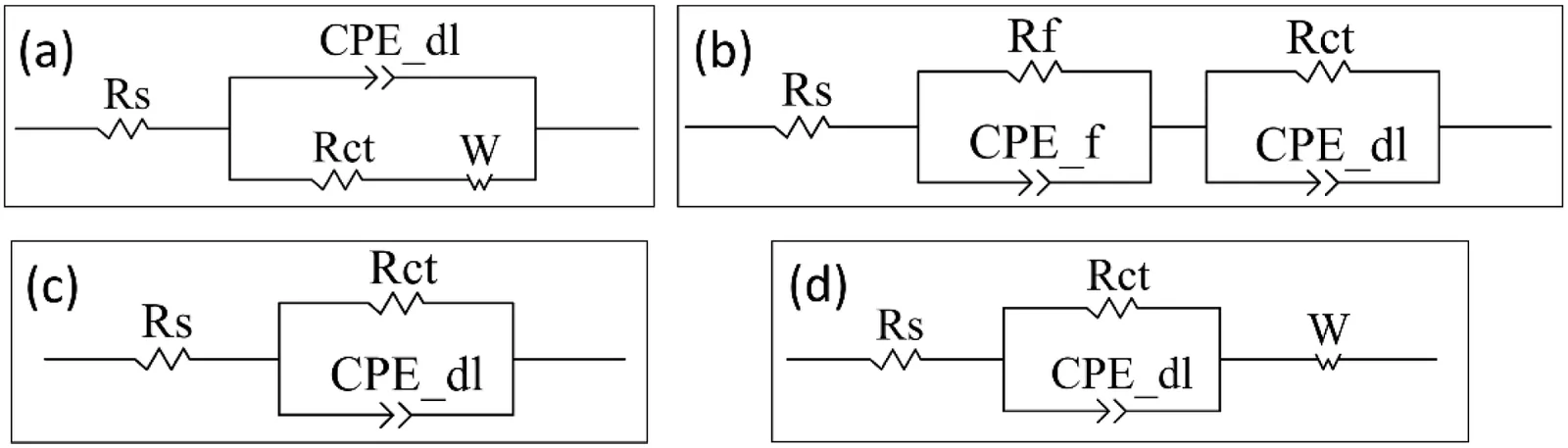
Equivalent fitting circuits for EIS data: (a) 20 °C immersion with corrosion products retained; (b) 20 °C salt spray with corrosion products retained; (c) 20 °C immersion with corrosion products removed, original sample, and −60 °C immersion sample; (d) 20 °C salt spray with corrosion products removed.

**Table 3:** Parameters of electrochemical impedance spectroscopy fitting circuit

Sample type	*R*_ct_ (Ω)	CPE_Y_0_	CPE_n	Note
Initial	1686.00	0.0004	0.7554	
20 °C immersion	630.50	0.0020	0.7005	Corrosion products retained
	1121.00	0.0011	0.8466	Corrosion products removed
20 °C salt spray	47.88	0.0069	0.5758	Corrosion products retained
	900.00	0.0010	0.8492	Corrosion products removed
−60 °C immersion	794.10	0.0014	0.6602	Corrosion products retained
	1176.00	0.0004	0.6568	Corrosion products removed

It can be seen from Table 3 that the salt spray sample with corrosion products retained has the smallest *R*_ct_, the largest CPE_Y_0_ and the smallest CPE_n, indicating that this sample has the worst corrosion resistance, the roughest surface and the most severe corrosion. Further comparison shows that after removing the corrosion products of immersion and salt spray samples, the charge transfer resistance *R*_ct_ increases significantly and the admittance constant CPE_Y_0_ decreases significantly, which further proves that the corrosion products have no protective effect on the substrate, but instead reduce the corrosion resistance of the substrate and accelerate substrate corrosion. A combined analysis of the EIS and polarization results shows that, after removal of the corrosion products, the corrosion resistance of all samples improved markedly relative to their as-exposed state, although the extent of recovery varied with the corrosion environment. For the 20 °C salt-spray sample, both *R*_ct_ (900.00 Ω) and *i*_corr_ (16.530 µA cm^−2^) remained significantly inferior to those of the initial sample, indicating that the damage caused by salt-spray corrosion was the most severe and could not be fully eliminated. For the immersion samples, although *i*_corr_ decreased to a level comparable to or lower than that of the initial sample, *R*_ct_ (1121.00 Ω and 1176.00 Ω) remained appreciably lower than that of the initial sample (1686.00 Ω), suggesting that the microstructural damage caused by immersion corrosion was only partially recovered.

### 3.5 Grain boundary and dislocation defect analysis after corrosion by EBSD

Fig. 8 shows the grain boundary distribution of samples under different corrosion conditions. Generally, grain boundary rotation angles are divided into two categories: low-angle grain boundaries (LAGB, 3°–15° misorientation) and high-angle grain boundaries (HAGB, >15° misorientation).^27^ As shown in Fig. 8a, c and e, all samples under different corrosion conditions are dominated by HAGB. Combined with the statistical results in Fig. 8g and h, the 20 °C salt spray sample has the highest HAGB proportion (69.4%) and grain boundary density (0.42 µm^−1^). HAGB, characterized by disordered atomic arrangements and high interfacial energy, serve as preferential sites for corrosive attack, which is consistent with the maximum corrosion rate of the salt spray sample. It should be emphasised that this correlation is established among specimens compared at the same temperature; across temperatures, the corrosion rate is governed primarily by temperature-dependent kinetics rather than by grain-boundary character.

**Fig. 8:**
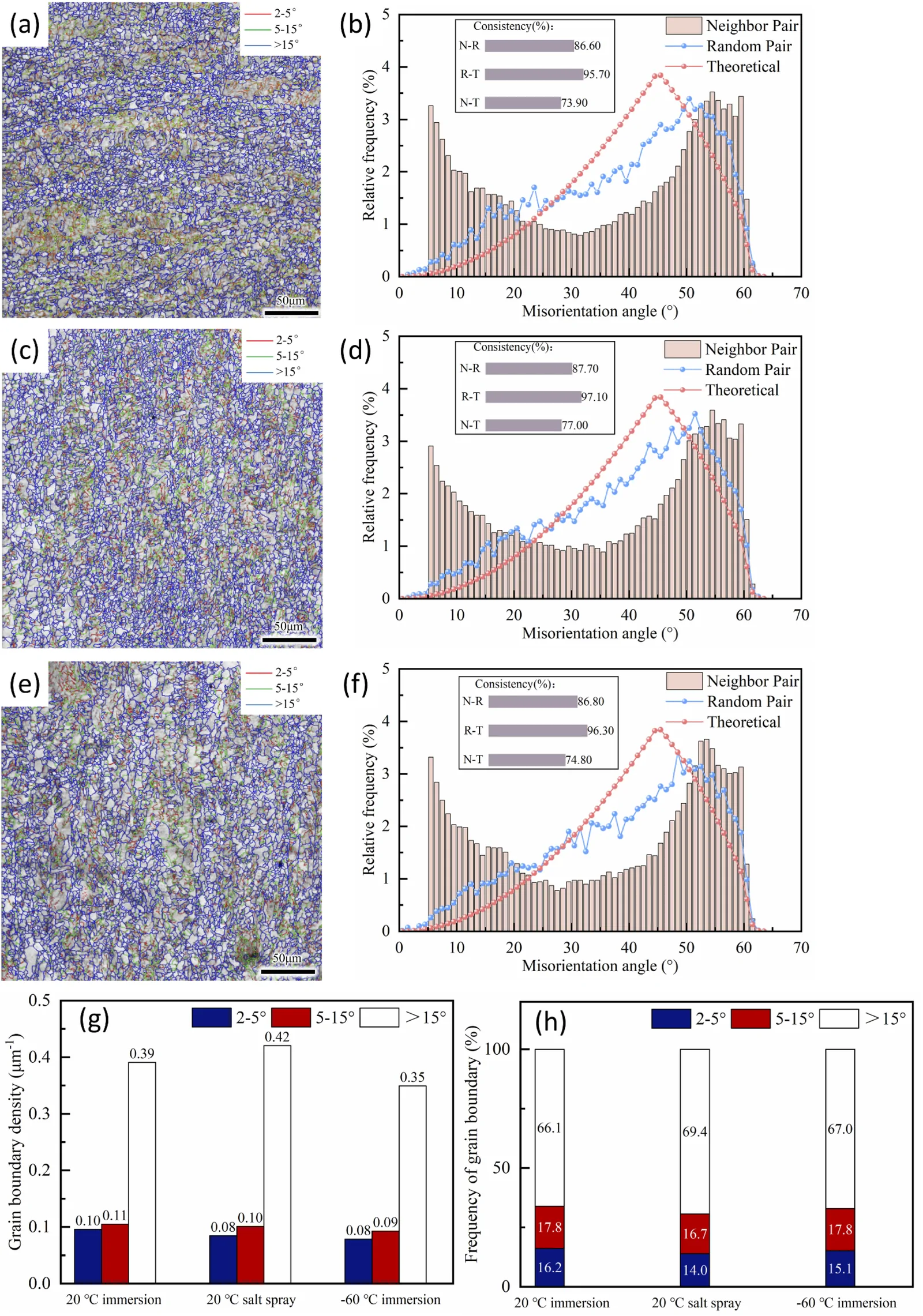
Grain boundary characteristics of FH40 under different corrosion conditions: (a, c and e) grain boundary orientation distribution and (b, d and f) misorientation distribution of the 20 °C immersed, 20 °C salt spray, and −60 °C immersed samples, respectively; (g) comparison of grain boundary density; (h) comparison of grain boundary orientation proportion.

The neighbor pair misorientation distribution in Fig. 8b, d and f reflects the local orientation gradient within grains and grain boundary characteristics, while the random pair misorientation distribution represents the overall orientation characteristics of the material. The theoretical-Mackenzie curve is the theoretical misorientation distribution of cubic polycrystalline materials with completely random orientations, which serves as a reference for evaluating crystallographic texture. As shown in Fig. 8b, d and f and the corresponding correlation analysis results, the random misorientation distributions of specimens under all three conditions exhibited correlation coefficients greater than 95% with the Mackenzie theoretical baseline (R–T), indicating that the misorientation distribution of all samples is highly consistent with the theoretical random distribution without obvious deviation. These results indicate that no significant preferred grain orientation was present in FH40 steel under any of the corrosion conditions examined, and that the differences in corrosion behavior across environments had a limited effect on the evolution of the crystallographic texture of FH40.

Fig. 9a, d and g presents the inverse pole figures (IPF-Z_0_) in the normal direction of samples under different corrosion conditions. The results show that no obvious preferred grain orientation is observed in all samples, which is consistent with the statistical results in Fig. 8. Fig. 9b, e and h shows the statistical histogram of coincidence site lattice (CSL) grain boundaries. Within the CSL model, the *Σ* value is a key parameter reflecting the volume ratio between coincidence lattice unit and matrix lattice unit. Grain boundaries can be classified according to *Σ* values: grain boundaries with *Σ* ≤ 29 have high coincidence site density, low interfacial energy and ordered structure, while grain boundaries with *Σ* > 29 have low coincidence site density, high interfacial energy and high susceptibility to defect initiation. This classification is widely used for the quantitative evaluation of grain boundary energy.^28^ Comparative analysis shows that the 20 °C salt spray sample exhibited the highest proportion of high-*Σ* grain boundaries (*Σ* > 29) at 5.72%, followed by 5.44% for the 20 °C immersed sample and 5.38% for the −60 °C immersed sample. This indicates that the grain boundary structure of FH40 is most severely damaged under 20 °C salt spray conditions. High-*Σ* grain boundaries, possessing sparse coincidence site density, elevated energy, and disordered structures, serve as preferential pathways for the penetration of corrosive media. In contrast, low temperature at −60 °C inhibits corrosion kinetics and maintains relatively stable grain boundary structure.

**Fig. 9:**
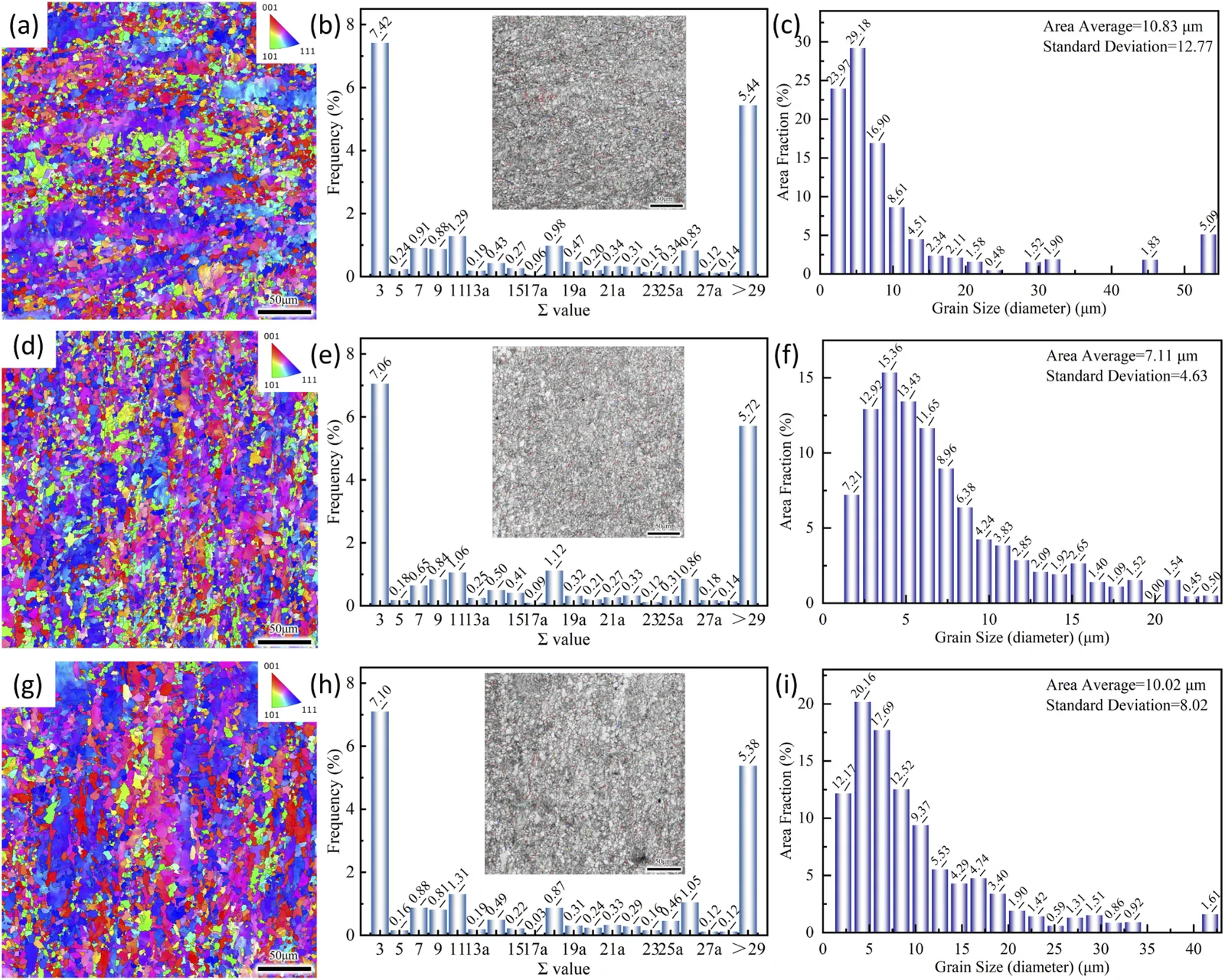
Inverse pole figures, CSL grain boundary distribution, and grain size distribution of FH40 under different corrosion conditions: (a–c) 20 °C immersed, (d–f) 20 °C salt spray, and (g–i) −60 °C immersed samples.

Fig. 9c, f and i shows the grain size distribution of all samples. The grain sizes of all samples are concentrated in the range of 2 µm to 20 µm. In terms of standard deviation, the grain size distribution of 20 °C and −60 °C immersed samples is more dispersed, while the 20 °C salt spray sample shows the most concentrated grain size distribution with the minimum average grain size of 7.11 µm. The apparent reduction in the measured grain size of the salt-spray specimen is consistent with the grain-boundary evolution shown in Fig. 8, 9b, e and h. Under dry–wet cyclic salt-spray conditions, sustained ingress of high-concentration chloride promotes preferential grain-boundary dissolution, which fragments and exposes finer surface grains and increases the measured HAGB density. The higher density of grain-boundary paths provides abundant corrosion-active sites, which further promotes corrosion development, suggesting a self-accelerating coupling between grain-boundary attack, the apparent reduction in grain size, the increase in grain-boundary density and the acceleration of corrosion, consistent with the highest corrosion rate of this condition.

Fig. 10a–i shows the grain orientation spread (GOS) and geometrically necessary dislocation (GND) density distribution of all samples. The GOS contour maps (Fig. 10a, d and g) show that the 20 °C salt spray sample has the maximum GOS value of 13.86°, which is higher than 11.77° of the 20 °C immersed sample and 11.04° of the −60 °C immersed sample. This indicates that severe grain boundary dissolution and fragmentation occur in the salt spray environment, leading to obvious grain orientation gradient. According to GND statistical results (Fig. 10c, f and i), the average GND density of the 20 °C immersed sample is 1.90 × 10^14^ m^−2^, which is the highest, followed by the 20 °C salt spray sample with 1.74 × 10^14^ m^−2^ and the −60 °C immersed sample with 1.70 × 10^14^ m^−2^. GND density reflects the level of dislocations induced by plastic deformation. Grain boundary dissolution in salt spray environment releases partial dislocation stress,^29^ leading to low average GND density. In contrast, sustained dislocation accumulation occurs during slow and uniform immersion corrosion, resulting in higher GND density. Low temperature suppresses atomic diffusion and dislocation motion, maintaining the dislocation density at a relatively low level under −60 °C conditions.

**Fig. 10:**
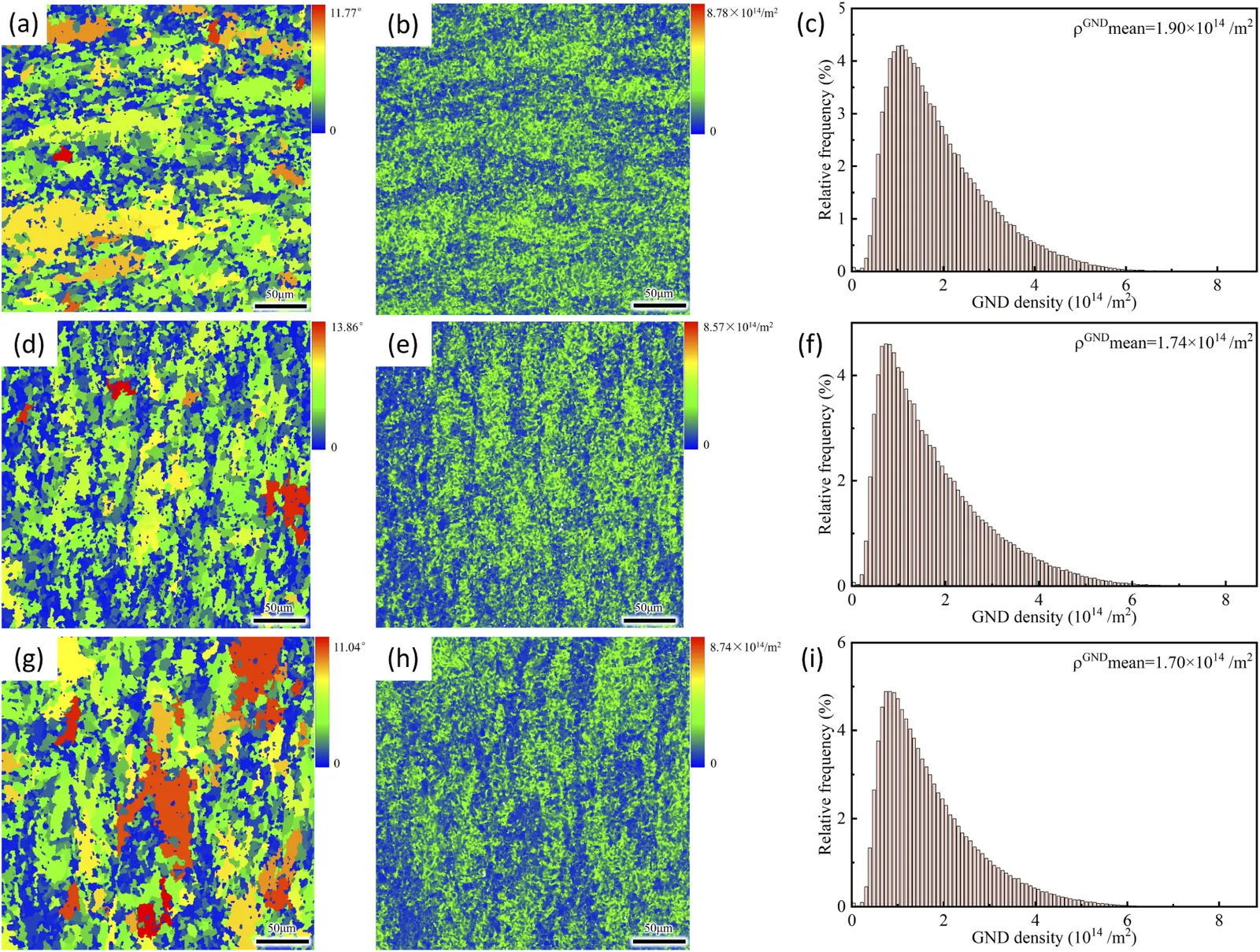
GOS distribution, GND density distribution, and GND histogram of FH40 under different corrosion conditions: (a–c) 20 °C immersed, (d–f) 20 °C salt spray, and (g–i) −60 °C immersed samples.

Overall, the corrosion damage under salt spray is dominated by grain-boundary dissolution with high GOS values, whereas immersion corrosion is accompanied by significant dislocation accumulation with a high GND density. The evolution of microstructural defects differs markedly among the corrosion conditions, consistent with the differences in corrosion rate and morphology.

### 3.6 Element distribution analysis of corrosion products

Fig. 11 shows the EDS mapping results of FH40 immersion and salt spray corrosion samples with corrosion products retained. It can be seen from Fig. 11a–f that the contents of elements other than C, Fe, O and Cl are relatively low in the corrosion products of FH40 samples under immersion and salt spray conditions. Furthermore, as C originates primarily from the sealing epoxy resin and surface contamination, it significantly dilutes the original mass fractions of Fe, O and Cl. To eliminate such interference and accurately compare the relative composition of corrosion products at different positions, the mass fractions of Fe, O, and Cl were renormalized to 100% for subsequent analysis, as presented in Fig. 11e.

**Fig. 11:**
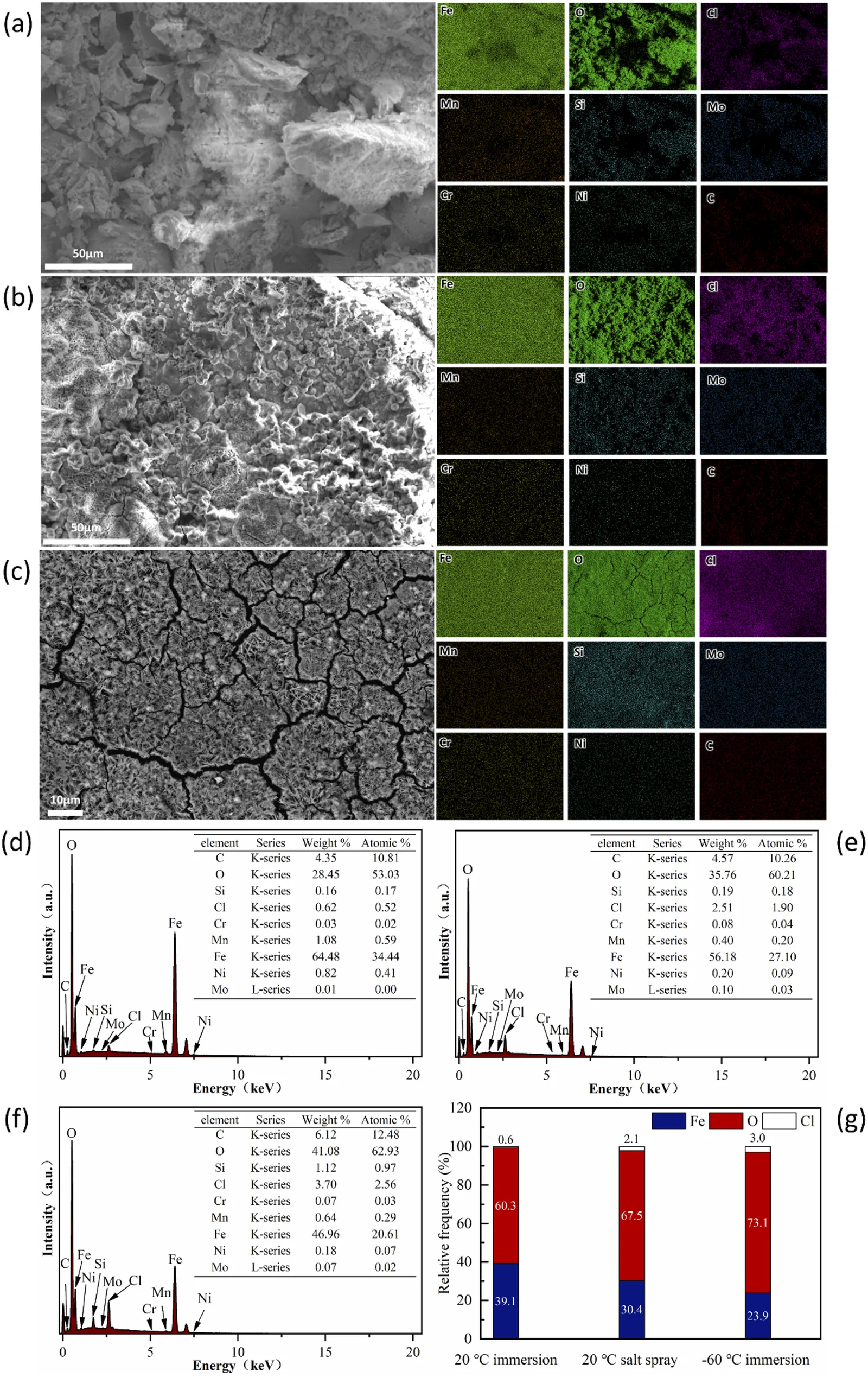
EDS mapping results of FH40 samples with corrosion products retained under three conditions: (a) 20 °C immersion, (b) 20 °C salt spray, and (c) −60 °C immersion; (d–f) corresponding semi-quantitative element contents, respectively; (g) semi-quantitative normalized element distribution.

Combined with the semi-quantitative element content results (Fig. 11e), it can be seen that under 20 °C condition, the Cl element content in the corrosion products of the salt spray sample is significantly higher than that of the immersion sample, which is also the reason why the mass loss and corrosion rate of the salt spray sample are higher than those of the immersion sample. Because the preferential accumulation of Cl^−^ within the corrosion products elevated the local Cl^−^ concentration, the erosion of Cl^−^ causes the dissolution of local corrosion products,^15^ inducing the formation of micropores and channels, making the salt spray corrosion products more porous and loose, providing contact channels for Cl^−^-containing electrolyte to reach the steel substrate directly, continuously accelerating the anodic dissolution of the substrate and further accelerating corrosion.

It can be seen from Fig. 11e that the Cl element content in the corrosion products of the −60 °C immersion sample is significantly higher than that of the 20 °C immersion sample, but its corrosion rate and corrosion layer thickness are much lower than those of the latter. Different from the 20 °C salt spray and immersion conditions, the increase of Cl element content in the corrosion products here is not accompanied by the increase of corrosion rate. This is mainly because the extreme low temperature (−60 °C) significantly inhibits the kinetic process of the corrosion reaction and changes the migration mode and action mechanism of Cl^−^: the water molecule activity is greatly reduced in the low-temperature environment, leading to a sharp reduction in ion migration rate,^30^ and Cl^−^ is difficult to quickly penetrate the corrosion product layer to reach the substrate surface,^31^ so the high content of Cl^−^ in the corrosion products is mostly bound to the surface of corrosion products and cannot participate in the dynamic corrosion electrochemical reaction.

Fig. 12 presents the EDS results of transverse cross-sections of FH40 samples after immersion and salt spray corrosion, aiming to reveal the differences in elemental distribution inside corrosion product layers. A comparison of the normalised O content (Fig. 11e and 12g) reveals that the proportion of surface O atoms (60.3–73.1%) under the three corrosion conditions is significantly higher than that in the internal cross-section (48.0–59.4%), with a relative decrease of 8–34%. This indicates a distinct oxygen concentration gradient within the corrosion product layer from the surface to the interior. The difference in oxygen content between the outer and inner layers is sufficient to form an oxygen concentration cell, driving anodic dissolution of the inner matrix and accelerating the corrosion process. This further demonstrates that the corrosion products of FH40 in this corrosive environment do not provide protection to the substrate. Compared with Fig. 11, the semi-quantitative results of each element in Fig. 12 show a significant jump in the proportion of C, which is because the resin used in making cross-section samples is rich in C and is included in the EDS collection, so the proportion of C in Fig. 12 rises sharply. It can be seen from the mapping results in Fig. 12a–f that the interior of the corrosion products of immersion and salt spray samples is still dominated by Fe and O, but the O/Fe ratio in the cross-section is significantly lower than that in the surface layer (Fig. 11), indicating that there is an obvious oxygen gradient between the inner and outer layers of the corrosion products of FH40 in the simulated marine corrosion environment, which can form an oxygen concentration cell and drive the anodic dissolution of the inner substrate, which further proves that the corrosion products of FH40 in this corrosion environment have no protective effect on the substrate. The line scan results in Fig. 12h–j show that the Cl^−^ content in the interior of the two corrosion products is similar, and only the surface content (Fig. 11d and e) is significantly different, which indicates that the high Cl^−^ on the surface is the decisive factor for the higher corrosion rate in the salt spray environment.

**Fig. 12:**
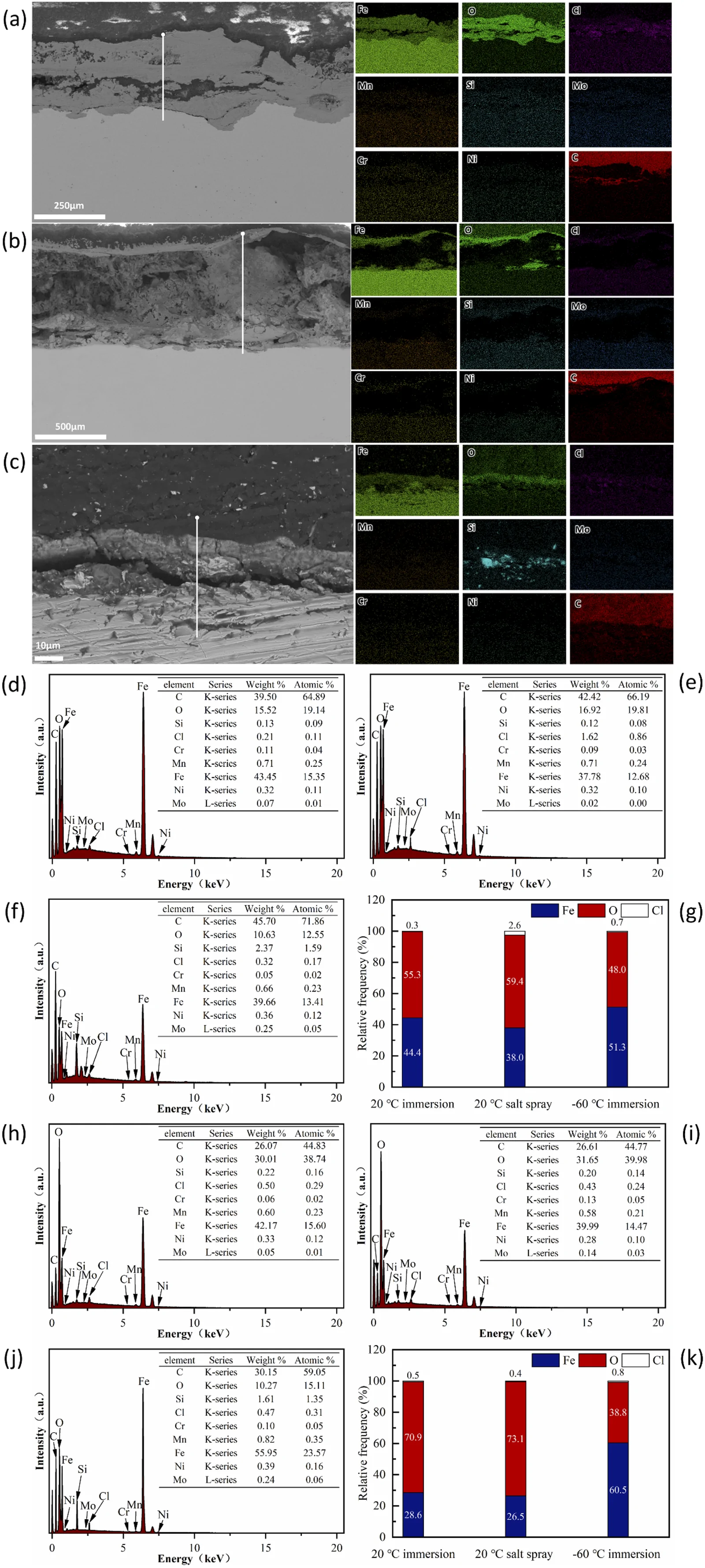
Cross-sectional EDS analysis of FH40 corrosion samples: (a–c) EDS maps of the 20 °C immersion, 20 °C salt spray, and −60 °C immersion cross-sections, respectively; (d–f) semi-quantitative element contents of the maps in (a–c); (g) normalized element distribution of the maps; (h–j) semi-quantitative element contents from line scans across the cross-sections in (a–c); (k) normalized element distribution of the line scans.

A comparison of the EDS results for the immersion cross-section at 20 °C and −60 °C (Fig. 12g and k) reveals that the distribution of Cl^−^ within the corrosion layer is essentially equivalent at both temperatures. This distribution pattern clearly indicates that low temperatures merely lead to the enrichment and retention of Cl^−^ in the surface layer of the corrosion layer (Fig. 11e), and a sharp decrease in ion migration rates,^30^ making it difficult for Cl^−^ to penetrate the corrosion product layer and reach the substrate surface,^31^ ultimately significantly inhibiting the electrochemical corrosion process and reducing the overall corrosion rate.

### 3.7 XRD analysis of corrosion products

Fig. 13 shows the XRD results of the corrosion products of FH40 immersion and salt spray corrosion samples. In comparison, the corrosion products of the 20 °C immersion sample are only γ-FeOOH and Fe(OH)_3_, while the corrosion products of the salt spray sample additionally detect α-FeOOH and β-FeOOH, and the corrosion products of the −60 °C immersion sample are mainly α-FeOOH and γ-FeOOH. In terms of microscopic morphology, Fe(OH)_3_ mainly exists in crystalline form, and its microscopic morphology in neutral environment is mainly granular;^32^ α-FeOOH is mainly granular or acicular;^33–35^ β-FeOOH is mainly rod-like;^34–36^ γ-FeOOH is mainly acicular or flaky crystals,^34,35,37^ which is consistent with Fig. 3 in this paper. It can be found from Fig. 13b that the XRD pattern of the salt spray corrosion products exhibited substantial noise and peak broadening, indicating poor crystallinity and a high amorphous/nanocrystalline fraction, which means that the corrosion products of the salt spray sample are mainly amorphous/nanocrystalline.^38,39^ This is also an important reason why the weight loss rate and corrosion rate of the 20 °C salt spray sample are higher than those of the 20 °C immersion sample, because when the corrosion products are mainly amorphous/nanocrystalline, they are macroscopically porous and loose,^40^ and defect sites become the preferred adsorption or embedding channels for Cl^−^, O_2_ and H_2_O.^41,42^ Microcracks are generated in dry–wet cycling, which further increases the permeability, and Cl^−^, O_2_, H_2_O and others continuously penetrate to accelerate substrate dissolution.

**Fig. 13:**
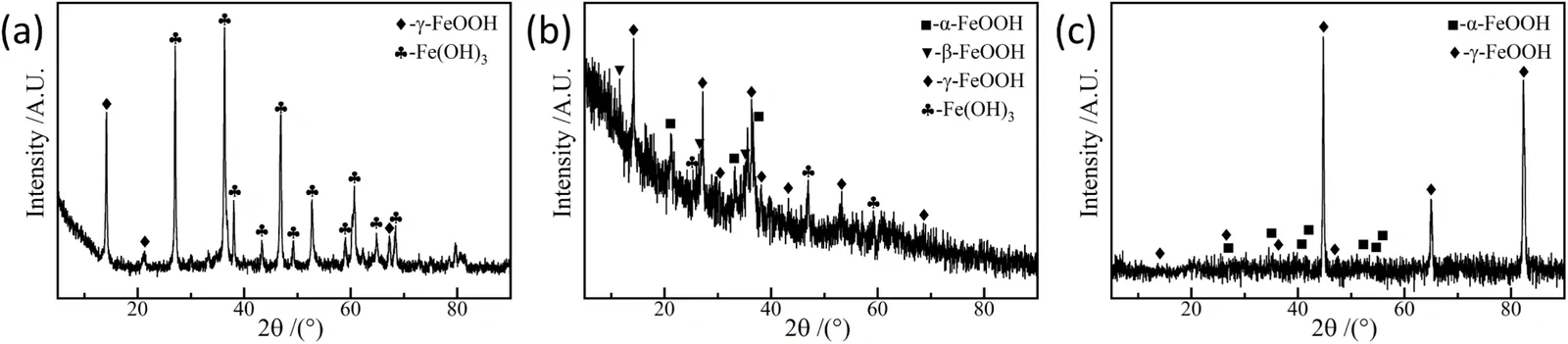
XRD patterns of corrosion products on FH40 samples after immersion and salt spray corrosion: (a) 20 °C immersion, (b) 20 °C salt spray, and (c) −60 °C immersion.

It can be seen from the XRD results in Fig. 13a and c that the corrosion products of the 20 °C immersion sample are mainly γ-FeOOH and Fe(OH)_3_, while Fe(OH)_3_ is not detected in the −60 °C immersion sample, and characteristic peaks of α-FeOOH appear instead. This difference is due to the regulation of low temperature on the formation and transformation of corrosion product phases: at room temperature, Fe^2+^ is rapidly oxidized and hydrolyzed to form the intermediate phase Fe(OH)_3_, which together with the metastable phase γ-FeOOH constitutes the corrosion products; at −60 °C, by contrast, the oxidation and hydrolysis kinetics of Fe^2+^ are strongly suppressed, so that Fe(OH)_3_ can hardly form and crystallise in quantity, while the transformation of the metastable phase towards the thermodynamically stable α-FeOOH is favoured. This result indicates that low temperature does not change the valence state distribution dominated by Fe^3+^, but regulates the kinetics to promote the evolution of corrosion products to the more stable α-FeOOH phase, and eliminates the unstable intermediate phase Fe(OH)_3_ at the same time.

### 3.8 Discussion

There are significant differences in corrosion mechanism and product composition of polar marine low-temperature steel FH40 under immersion and salt spray corrosion environments. 20 °C immersion is characterized by uniform dissolution accompanied by localized pitting corrosion, −60 °C immersion is typical localized pitting corrosion, while 20 °C salt spray presents a crater-like corrosion morphology with internal accumulation and edge deep etching (Fig. 4).

Under 20 °C condition, the surface of corrosion products of the salt spray sample is rougher, the interior is more porous and loose, and the thickness is higher (Fig. 2 and 3), which is closely related to the Cl^−^ enrichment on its surface (Fig. 11): the erosion of Cl^−^ causes the dissolution of local products and forms micropores and channels, providing channels for Cl^−^-containing electrolyte to reach the substrate directly; dry–wet cycling further induces microcracks and improves permeability, promoting the continuous penetration of Cl^−^, O_2_ and H_2_O to accelerate substrate dissolution. Although the thickness of corrosion products of the salt spray sample is 3–4 times that of the immersion sample (Fig. 3h and i), its mass loss and corrosion rate are 3–4 times higher than those of the immersion sample (Table 1), which indicates that there is no simple positive correlation between the thickness of corrosion products and the protective effect on the substrate, and the corrosion resistance cannot be judged only by the thickness of corrosion products.

The EBSD characterisation reveals the microstructural aspects of the environmental effects on the corrosion behaviour. HAGBs predominate in all specimens, and the 20 °C salt-spray specimen exhibits the highest HAGB fraction and grain-boundary density. Because HAGBs possess disordered atomic arrangements and high interfacial energy, they can act as preferential corrosion sites; under salt spray, sustained chloride attack and dry–wet cycling significantly aggravate grain-boundary dissolution. The salt-spray specimen also shows the highest high-*Σ* fraction and the smallest measured grain size, indicating the most severe grain-boundary damage among the three conditions. It should be emphasised, however, that this correlation between grain-boundary character and corrosion severity is established among specimens compared at the same temperature; across temperatures, the corrosion rate is governed primarily by temperature-dependent kinetics rather than by grain-boundary character, as evidenced by the nearly identical HAGB and high-*Σ* fractions—yet order-of-magnitude different corrosion rates—of the 20 °C and −60 °C immersion specimens. The inverse pole figures and misorientation distributions confirm that no pronounced texture developed in any specimen, so the differences in corrosion behaviour are not caused by texture evolution. The microstructural-defect analysis further reveals different damage signatures: salt-spray corrosion is characterised by severe grain-boundary damage and high GOS values, whereas immersion corrosion leads to sustained dislocation accumulation. The low temperature suppresses dislocation motion and microstructural degradation, resulting in low corrosion activity.

Combined with XRD and morphology analysis, the 20 °C immersion products are mainly γ-FeOOH and Fe(OH)_3_, corresponding to cluster-like cauliflower particles and local flaky protrusions (Fig. 3a and b); the 20 °C salt spray products show the coexistence of flaky, rod-like and granular shapes, corresponding to γ-FeOOH, β-FeOOH and α-FeOOH/Fe(OH)_3_ respectively (Fig. 3d–f); the −60 °C immersion products are acicular α-FeOOH and flaky γ-FeOOH (Fig. 3j and k), reflecting the regulatory effect of low temperature on phase evolution. From the perspective of thermodynamic Gibbs free energy, the evolution law of different phases originates from the competitive effect between their thermodynamic stability and reaction kinetics: in the 20 °C corrosion environment, the thermal motion energy is sufficient, and the overall kinetic rate of the corrosion reaction is fast. Fe^2+^ has no time to complete atomic rearrangement to cross the high energy barrier to generate α-FeOOH, but directly transitions from the high energy state and precipitates into the metastable phase γ-FeOOH and the intermediate phase Fe(OH)_3_ with lower activation energy and easier precipitation,^43^ as shown in eqn (4)–(6). This reflects the evolution law of kinetic priority at room temperature, that is, the metastable phase with faster generation rate and lower energy barrier precipitates first; in the extreme low temperature environment of −60 °C, although the intensity of molecular thermal motion is greatly reduced and the overall reaction rate is significantly inhibited, the system shows a significant thermodynamic tendency to be stable. The low temperature environment greatly reduces the ion migration rate, making the corrosion reaction have sufficient time for atomic-level recombination and dehydration, overcoming the energy barrier and transforming into α-FeOOH with the lowest Gibbs free energy and the most thermodynamic stability, as shown in eqn (7). Therefore, the phenomenon that Fe(OH)_3_ is not found and α-FeOOH is detected in the −60 °C sample in the XRD results is essentially a thermodynamic result of the extreme low temperature strengthening the convergence of the system to the lowest energy state.

2 Anode: Fe → Fe^2+^ + 2e^−^, Fe^2+^ → Fe^3+^ + e^−^

3 Cathode: O_2_ + 2H_2_O + 4e^−^ → 4OH^−^

4 20 °C condition: Fe^2+^ + 2OH^−^ → Fe(OH)_2_

5 4Fe(OH)_2_ + 2H_2_O + O_2_ → 4Fe(OH)_3_

6 Fe(OH)_3_ → α/β/γ-FeOOH + H_2_O

7 −60 °C condition: Fe^3+^ + 3OH^−^ → α/γ-FeOOH + H_2_O

Electrochemical tests show that after removing the rust layer (Tables 2 and 3), the corrosion current density of each sample decreases significantly and the charge transfer resistance increases significantly, with the salt spray sample showing the largest recovery (*R*_ct_ increasing from 47.88 Ω to 900.00 Ω), but still lower than the original substrate, indicating that the micro-defects caused by corrosion will continuously weaken the corrosion resistance of the material, and the corrosion products of FH40 formed in immersion and salt spray environments will not have a protective effect on the substrate, but instead accelerate corrosion. The EDS mapping results (Fig. 11e and 12g) further reveal that there is an obvious oxygen gradient between the inner and outer layers of the corrosion products, which is easy to form an oxygen concentration cell and drive the anodic dissolution of the inner substrate, which is the key electrochemical mechanism for the products to accelerate corrosion.

In terms of the influence of low temperature, although the frozen NaCl solution at −60 °C still has conductivity and corrosivity, the corrosion rate and corrosion layer thickness are an order of magnitude different from those at 20 °C (Table 1 and Fig. 3h, m), showing a significant corrosion inhibition effect. Low temperature slows down corrosion through a dual mechanism: on the one hand, the activity of water molecules and the ion migration rate drop sharply, resulting in the enrichment and retention of Cl^−^ on the surface of the corrosion product layer (Fig. 11 and 12), making it difficult for Cl^−^ to penetrate through to the substrate and engage in the electrochemical reaction; on the other hand, low temperature promotes more complete oxidation of Fe^2+^ through kinetic regulation, facilitating the transformation of the metastable Fe(OH)_3_ phase into the thermodynamically stable α-FeOOH (Fig. 13a and c), which forms a more compact product layer that further impedes the corrosion process.

Fig. 14 is a schematic diagram of the corrosion mechanism of FH40 steel under different corrosion environments, which can further clarify the corrosion path and dominant mechanism of FH40 steel under different corrosion environments. In the 20 °C immersion environment (Fig. 14a), uniform dissolution and localized pitting corrosion occur synergistically, and the corrosion products are mainly γ-FeOOH and Fe(OH)_3_, presenting cluster-like and flaky morphologies, reflecting the precipitation law of metastable phases with kinetic priority at room temperature. In the 20 °C salt spray environment (Fig. 14b), dry–wet cycling and Cl^−^ enrichment act together: salt spray deposition makes a large amount of Cl^−^ accumulate on the surface of the rust layer, inducing local product dissolution and forming micropores and channels, resulting in a crater-like morphology with “internal accumulation and edge deep etching”. Although the corrosion product layer is thick, it is porous and loose, unable to provide effective protection; on the contrary, it accelerates substrate dissolution due to the oxygen concentration cell effect. In the −60 °C low-temperature immersion environment (Fig. 14c), the water molecule activity and ion migration rate decrease significantly, Cl^−^ is enriched and retained on the surface of the rust layer, difficult to participate in the interface reaction. The dual inhibition effect makes the corrosion rate and rust layer thickness decrease significantly, the corrosion process is inhibited to localized pitting corrosion, and the products are mainly thermodynamically stable α-FeOOH.

**Fig. 14:**
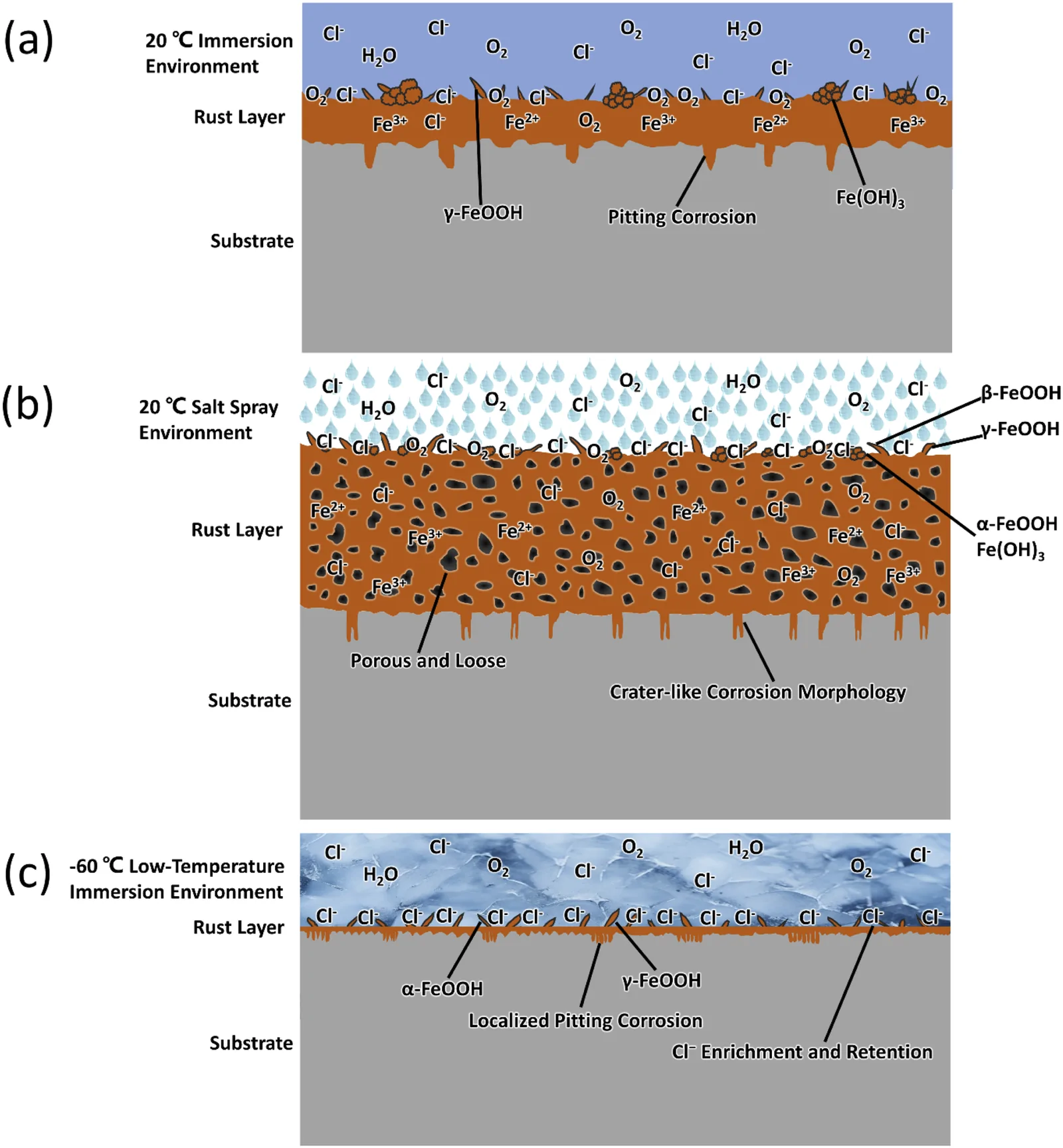
Schematic illustration of the corrosion mechanism of FH40 steel in different environments: (a) 20 °C immersion, (b) 20 °C salt spray, and (c) −60 °C immersion.

On the whole, the three groups of mechanism models systematically reveal the synergistic effect of temperature and corrosion medium supply mode: corrosion at room temperature is dominated by kinetics, and the salt spray environment amplifies the local corrosion effect through the continuous transport of Cl^−^ and dry–wet cycling; low temperature significantly slows down the corrosion process by inhibiting the mass transfer process and promoting the transformation of products to thermodynamically stable phases.

## 4 Conclusions

This work systematically compared the corrosion behaviour of FH40 polar marine low-temperature steel under immersion and neutral salt-spray conditions at 20 °C and −60 °C. The main conclusions are as follows:

(1) The corrosion mechanism is synergistically governed by the exposure environment and temperature. Immersion at 20 °C is dominated by uniform dissolution with localised pitting; immersion at −60 °C is confined to typical localised pitting; and salt spray at 20 °C produces a crater-like morphology with internal accumulation and deep edge attack, driven by dry–wet cycling and Cl^−^ enrichment.

(2) The corrosion products formed on FH40 are non-protective under all three conditions. The oxygen-concentration gradient between the outer and inner layers of the rust establishes an oxygen concentration cell that sustains the anodic dissolution of the substrate. Although the rust layer formed under salt spray is 3–4 times thicker than that formed under immersion, the mass loss and corrosion rate are also 3–4 times higher, confirming that rust-layer thickness alone is not a reliable indicator of protectiveness.

(3) EBSD characterisation shows that HAGBs predominate in all specimens and that the salt-spray specimen exhibits the highest HAGB fraction, grain-boundary density and high-*Σ* boundary fraction, together with the smallest measured grain size, consistent with its most severe corrosion attack at ambient temperature; immersion corrosion is instead accompanied by sustained dislocation accumulation. Across temperatures, the corrosion rate is governed primarily by temperature-dependent kinetics rather than by grain-boundary character.

(4) Low temperature (−60 °C) suppresses the corrosion rate and the rust-layer thickness by approximately one order of magnitude through a dual mechanism: suppression of water activity and ion migration, which immobilises Cl^−^ at the rust surface, and kinetic regulation of the product evolution, which favours the thermodynamically stable α-FeOOH at the expense of the metastable Fe(OH)_3_ intermediate.

These findings provide a theoretical basis for predicting the service life and designing protective strategies for polar marine vessels operating in harsh Arctic environments.

## Author contributions

Liangliang Hu: conceptualization, methodology, investigation, formal analysis, data curation, visualization, writing – original draft. Dongsheng Wang: conceptualization, supervision, funding acquisition, writing – review & editing. Juan Zhao: investigation, formal analysis. Yitao Wang: investigation, methodology. Xuhui Gong: investigation, data curation. Penglong Ding: investigation, formal analysis. Xueting Chang: supervision, funding acquisition, writing – review & editing, project administration.

## Conflicts of interest

The authors declare that they have no known competing financial interests or personal relationships that could have appeared to influence the work reported in this paper.

## Data Availability

The data that support the findings of this study are available from the corresponding author upon reasonable request.
